# The mTOR inhibitor everolimus in combination with azacitidine in patients with relapsed/refractory acute myeloid leukemia: a phase Ib/II study

**DOI:** 10.18632/oncotarget.13699

**Published:** 2016-11-29

**Authors:** Peter Tan, Ing Soo Tiong, Shaun Fleming, Giovanna Pomilio, Nik Cummings, Mark Droogleever, Julie McManus, Anthony Schwarer, John Catalano, Sushrut Patil, Sharon Avery, Andrew Spencer, Andrew Wei

**Affiliations:** ^1^ Malignant Haematology and Stem Cell Transplantation Service, Alfred Hospital, Melbourne, Australia; ^2^ Australian Centre for Blood Diseases, Monash University, Melbourne, Australia; ^3^ Department of Pathology, Alfred Hospital, Melbourne, Australia; ^4^ Faculty of Medicine, University of Amsterdam, Amsterdam, The Netherlands; ^5^ Eastern Health Clinical School, Monash University, Box Hill, Australia; ^6^ Clinical Haematology, Frankston Hospital, Frankston, Australia

**Keywords:** acute myeloid leukemia, everolimus, azacitidine, mTOR, clinical trial

## Abstract

Therapeutic options are limited in relapsed/refractory acute myeloid leukemia (AML). We evaluated the maximum tolerated dose (MTD) and preliminary efficacy of mammalian target of rapamycin (mTOR) inhibitor, everolimus (days 5–21) in combination with azacitidine 75 mg/m^2^ subcutaneously (days 1–5 and 8–9 every 28 days) in 40 patients with relapsed (*n* = 27), primary refractory (*n* = 11) or elderly patients unfit for intensive chemotherapy (*n* = 2). MTD was not reached following everolimus dose escalation (2.5, 5 or 10 mg; *n* = 19) to the 10 mg dose level which was expanded (*n* = 21). Major adverse events (grade > 2) were mostly disease-related: neutropenia (73%), thrombocytopenia (67%), mucositis (24%) and febrile neutropenia (19%). Overall survival (OS) of the entire cohort was 8.5 months, and overall response rate (ORR; including CR/CRi/PR/MLFS) was 22.5%. Furthermore, a landmark analysis beyond cycle 1 revealed superior OS and ORR in patients receiving 2.5 mg everolimus with azoles, compared to those without azoles (median OS 12.8 vs. 6.0 months, *P* = 0.049, and ORR 50% vs. 16%, *P* = 0.056), potentially due to achievement of higher everolimus blood levels. This study demonstrates that everolimus in combination with azacitidine is tolerable, with promising clinical activity in advanced AML.

## INTRODUCTION

Mammalian target of rapamycin (mTOR) is a serine/threonine protein kinase regulating cell growth, proliferation, survival, autophagy, gene transcription and protein synthesis. Activation of the PI3K/AKT/mTOR signaling pathway is prevalent in acute myeloid leukemia (AML) [1, 2], and constitutive activation of AKT is associated with inferior survival in AML [3]. mTOR phosphoactivates downstream 4EBP1 and p70S6K. Its inhibition mediates cell cycle arrest through 4EBP1 dephophosphorylation, increased p27 and reduced cyclin D1 activity. Rapamycin-induced targeting of mTOR complex 1 (mTORC1) may result in paradoxical feedback hyperactivation of AKT [4, 5]. Although mTOR complex 2 (mTORC2) is thought to be rapamycin insensitive, it may be inhibited by higher doses of rapamycin, leading to AKT and ERK suppression [6]. Rapamycin derivatives, temsirolimus and everolimus, have been shown to inhibit both mTORC1 and mTORC2, leading to inhibition of AKT in AML [7].

Small molecule targeting of mTOR has been shown to selectively inhibit the clonogenic function of leukemic, but not normal progenitors, suggesting a potential role in modulating relapse from surviving progenitors after conventional cytotoxic approaches [8–10]. Although mTOR inhibitors have limited clinical activity in AML as monotherapy [8, 11–14], combination with cytotoxic approaches appears more promising. Everolimus combined with conventional 7+3 chemotherapy produced a complete remission (CR) rate of 68% in AML at first relapse that was correlated with the extent of plasma inhibition of phosphorylated p70S6K (P-p70S6K) [15]. Sirolimus in combination with mitoxantrone, etoposide and cytarabine in relapsed/refractory AML produced a response rate of 22% [16], and temsirolimus in combination with clofarabine salvage therapy produced a response rate of 21% [17].

In relapsed/refractory AML, hypomethylating agents characteristically produce CR rates of approximately 20% and OS outcomes of between 6–9 months [18–20]. Pre-clinical data in both solid cancers [21–25] and AML [26, 27] support the rationale for combined targeting of mTOR and gene methylation pathways. Synergism has been observed in some AML cell lines when these 2 classes of agents have been combined, with greater AKT suppression than observed with either agent alone [26]. Hypomethylating agents may also promote expression of mTOR pathway inhibitors, such as *PTEN* and *PPP2R2B* [23], *TSC2* [24, 27], and miR-34a [25].

To date, only one clinical study has combined an mTOR inhibitor and a hypomethylating agent in relapsed/refractory AML. Decitabine 20 mg/m^2^ was given on days 1–5 followed by first generation mTOR inhibitor rapamycin on days 6–25. Among 12 evaluable patients, the median survival was 4 months and only one CR was achieved. [29]. To determine if everolimus, a more potent mTOR inhibitor, would be tolerable and effective at augmenting the activity of hypomethylating agents in AML, we herein report a dose-finding study of azacitidine in combination with the rapamycin derivative everolimus in relapsed and refractory AML.

## RESULTS

### Study population

Between March 2010 and July 2011, forty patients were enrolled on the study, nineteen in phase I and 21 in phase II. Baseline patient data is shown in Table 1. The median age of the group was 64.8 years (range 17.7–78.5 years). Twenty-seven patients (67.5%) had relapsed (6 after prior allogeneic stem cell transplantation, and 5 relapsed < 6 months after the prior line of therapy), eleven (27.5%) had primary refractory, and 2 (5%) had previously untreated AML. Adverse risk karyotype was present in 13 patients (33%), secondary/therapy-related AML in 8 patients (23%), *IDH1/2* mutation in 15/35 (43%), and *FLT3*-ITD mutation in 7/37 (19%). Thirty-four patients completed at least 1 cycle of study therapy, with a median of 4 cycles (range 1–30) administered. Twenty-eight patients (70%) had prior exposure to high-dose cytarabine, with 18 patients (45%) having received 0–1 prior lines of therapy and 22 (55%) 2–3 prior lines of therapy.

**Table 1:** Baseline patient characteristics

Characteristics	Patients, *n* (%)
Age, years – median (range)	65 (17–78)
Sex	
Male	24 (60)
Female	16 (40)
Previous lines of therapy	
0	2 (5)
1	15 (37·5)
2	16 (40)
3	7 (17·5)
Previous allogeneic/autologous transplant	6/3 (22·5)
Relapsed AML	27 (67·5)
Early (≤ 6 months)	22
Late (> 6 months)	5
Refractory AML	12 (32)
ECOG PS	
0–1	35 (88)
2	5 (12)
AML karyotype	
Intermediate	25 (62·5)
Normal	13
Adverse	13 (32·5)
Complex	3
Monosomy 7/del(7q)	7
*MLL* rearrangements	2
t(3;3)	2
sAML/tAML	8/1 (22·5)
Bone marrow blast count, % – mean ± sd	41·4 ± 30·1

*Abbreviations*: AML, acute myeloid leukemia; ECOG PS, Eastern Cooperative Oncology Group Performance Score; *MLL*, mixed-lineage leukemia; sAML, secondary AML; tAML, therapy-related AML.

### Toxicity and maximum tolerated dose

Three patients withdrew early due to disease progression, leaving 37 patients evaluable for toxicity. Two patients developed a first cycle dose-limiting toxicity (DLT) during the phase I dose escalation phase. At the 2.5 mg everolimus dose level, one patient developed grade 3 mucositis, whereas at the 5 mg dose level another patient developed grade 3 septic arthritis. No DLTs were experienced at the 10 mg dose level and thus the maximum tolerated dose (MTD) was not defined. The 10 mg everolimus dose level was the recommended phase 2 dose.

The combination of everolimus and azacitidine was generally well tolerated, with the most common adverse events listed in Table 2, irrespective of causality. Beyond the initial cycle, two patients developed Common Terminology Criteria for Adverse Events (CTCAE) grade 5 invasive fungal infection in the setting of progressive AML. The most common first cycle non-hematologic adverse events were mucositis (*n* = 9, 24.3%), febrile neutropenia (*n* = 7, 18.9%), hemorrhage (*n* = 5, 13.5%), diarrhea (*n* = 5, 13.5%), fatigue (*n* = 4, 10.8%) and nausea (*n* = 4, 10.8%). The most common severe (CTCAE grade 3 or higher) non-hematologic toxicities beyond first cycle among 34 evaluable patients were infection (*n* = 11, 32.3%), febrile neutropenia (*n* = 11, 32.3%) and hemorrhage (*n* = 3, 8.8%). Hematologic toxicities were common, with 21/31 evaluable patients (67.7%) developing CTCAE grade 3 or 4 anemia, 11/15 patients (73.3%) developing grade 3 or 4 neutropenia and 14/21 (66.7%) developing grade 3 or 4 thrombocytopenia (Supplementary Table S2).

**Table 2:** Toxicities in the treated population^a^

Everolimus dose Adverse event, *n* (%)	All (*n* = 37)	2.5 mg (*n* = 6)		5 mg (*n*= 11)		10 mg (*n* = 20)	
		Gr. 1/2	Gr. > 2	Gr. 1/2	Gr. > 2	Gr. 1/2	Gr. > 2
Mucositis	9 (24.3)	2	1^b^	2		3	1
Febrile neutropenia	7 (18.9)		2				5
Bleeding	5 (13.5)					3	2
Diarrhea	5 (13.5)			3		1	1
Infection	5 (13.5)			1	1	1	2^c^
Fatigue	4 (10.8)			2		2	
Nausea	4 (10.8)	1				3	
Creatinine elevated	3 (8.1)			2		1	
Fever	3 (8.1)			1		2	
Sepsis	3 (8.1)		1		1^b^		1
Pain	2 (5.4)					2	
Dyspnea	2 (5.4)			1		1	
Cough	2 (5.4)			1		1	
Dental pain	1 (2.7)			1			
Deranged LFTs	1 (2.7)		1				
Injection site reaction	1 (2.7)			1			
Anorexia	1 (2.7)					1	
Osteoporosis	1 (2.7)						1
Rash	1 (2.7)					1	
Throat pain	1 (2.7)					1	

^a^Three patients did not complete the initial planned therapy due to disease progression and were not evaluable for toxicities. Toxicities in patients who received concomitant azole with everolimus (2·5 mg) were not shown but were not significantly different to the remaining patients.

^b^Dose-limiting toxicity was observed in 2 patients: one had grade 3 mucositis and another had grade 3 septic arthritis.

^c^Grade 5 invasive fungal infection was observed in 2 patients.

*Abbreviations*: Gr., grade; LFTs, liver function tests.

The majority of patients exiting the study did so due to disease progression or relapse (*n* = 29, 76.3%), including two early deaths from AML progression. Other reasons for study discontinuation included allogeneic hematopoietic stem cell transplantation (*n* = 5, 13.2%), deranged liver function tests due to graft versus host disease (*n* = 1) and metastatic melanoma (*n* = 1).

### Clinical outcome

**Figure 1:**
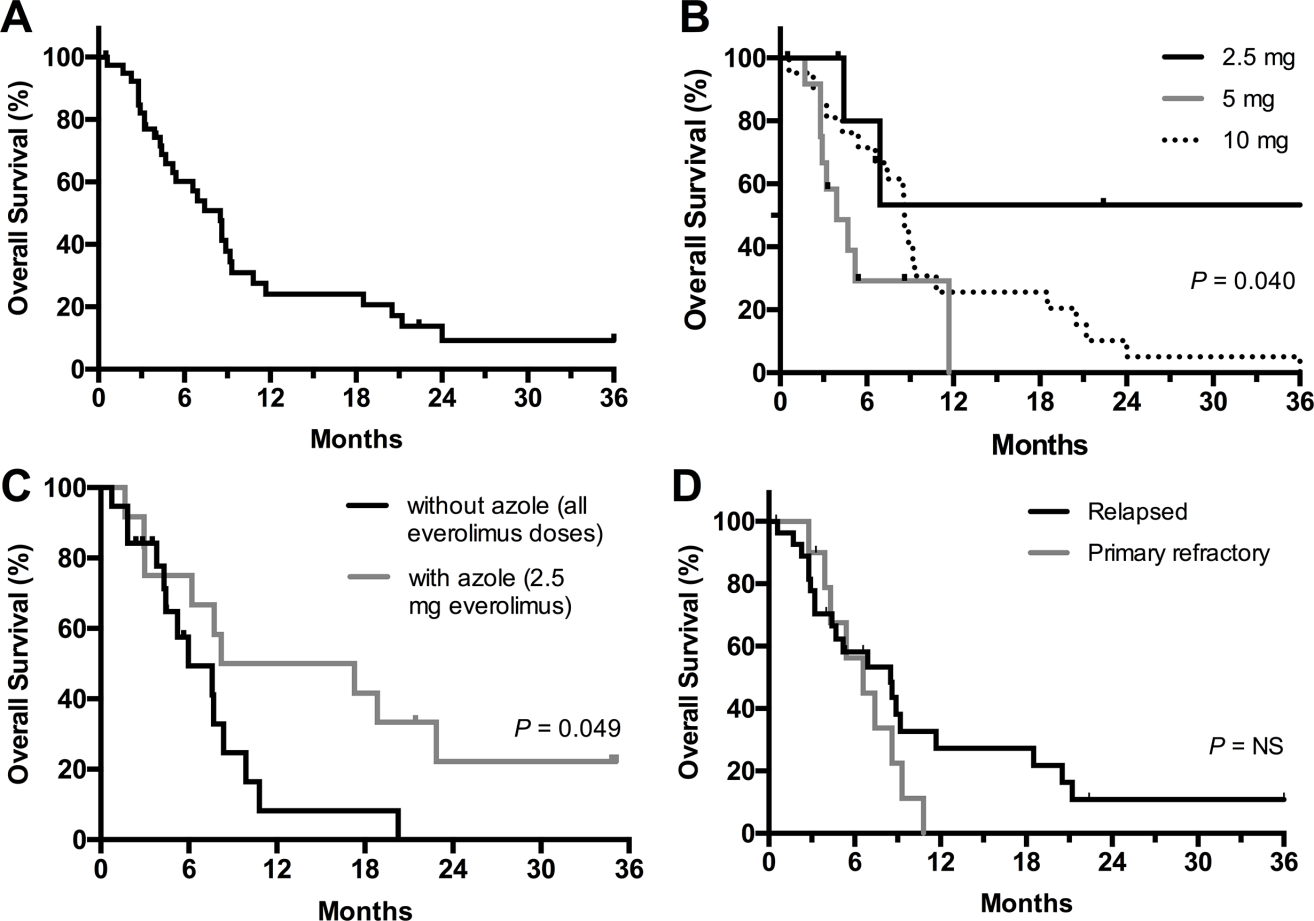
Overall survival of patients in the entire cohort (A) and according to everolimus dose cohort (B), everolimus with or without concurrent azole (C), and disease status (relapse or primary refractory) at study entry (D)

International Working Group (IWG) overall response rate was 9/40 (22.5%, Table 3). These included CR or CR with incomplete blood count recovery (CRi) in 5 (12.5%), one morphologic leukemia-free state (MLFS, 2.5%), and three partial remission (PR, 7.5%). The median relapse-free survival (RFS) among the responders was 4.1 months (range 0.8–36+ months). Median time to best response was 3 cycles (range 1–21). The median duration of follow up was 36 months for the survivors. The median OS for the entire cohort was 8.5 months (Figure 1A). Median OS according to the everolimus dose was not reached in the 2.5 mg cohort (*n* = 6), 3.9 months in the 5 mg cohort (*n* = 12), and 8.6 months in 10 mg cohort (*n* = 22) (Figure 1B).

**Table 3:** Summary of clinical responses

Responses in AML patients (*n* = 40)	Everolimus dose				Overall (%)
	2.5 mg (*n* = 3)	5 mg (*n* = 12)	10 mg (*n* = 13)	2.5 mg + azole^a^ (*n* = 12)	
CR/CRi		1		4	5 (12.5)
MLFS				1	1 (2.5)
PR		1	1	1	3 (7.5)
Overall (%)	0	2 (16.7)	1 (7.7)	6 (50)	9 (22.5)
Resistant disease^b^	3	7	10	6	26 (65)
Others^c^		3	2		5 (12.5)

^a^This cohort comprised 3 patients initially in the 2.5 mg “without-azole” cohort and 9 patients initially in the 10 mg expansion cohort with everolimus dose reduced to 2.5 mg from cycle 2 onwards in combination with an antifungal azole.

^b^3 patients in the 5 mg cohort did not have an evaluable bone marrow examination but had persistent leukemia in peripheral blood.

^c^2 patients in the 10 mg cohort died from disease progression before completion of therapy. 3 patients in the 5 mg cohort withdrew due to toxicities before completion of the initial therapy.

*Abbreviations*: CR, complete remission; CRi, complete remission with incomplete blood count recovery; MLFS, morphologic leukemia-free state; PR, partial remission

Three patients initially in the 2.5 mg cohort and 9 patients initially in the 10 mg cohort subsequently received azole antifungal prophylaxis from cycle 2 onwards, with the everolimus dose capped at 2.5 mg due to the higher drug levels expected from the interaction with CYP3A4 inhibitory azoles. A landmark analysis was performed on patients from cycle 2 day 1 (resulting in exclusion of 9 patients). Interestingly, ORR and OS were higher among patients who received everolimus and concurrent azoles, compared to those not receiving concurrent azoles: ORR 6/12 (50%) vs. 3/19 (16%, *P* = 0.056) and median OS 12.8 vs. 6.0 months (*P* = 0.049, Figure 1C), respectively.

Median OS for patients with baseline intermediate cytogenetic risk was 8.6 months versus 3.8 months for patients with adverse risk (*P* = 0.06). Median survival outcome was comparable for patients with relapsed and primary refractory AML (Figure 1D).

### Correlative studies

#### Molecular

Thirty-seven patients had cytogenetic and molecular abnormalities characterized and these were compared to the relative bone marrow blast reductions in 33 evaluable patients (Figure 3). Acknowledging the small patient numbers available, encouraging responses (at least 50% reduction in marrow blasts) were observed in patients with *FLT3*-ITD (3/6) and *MLL*-rearranged leukemia (2/2). Of note, 3/9 patients with complex or monosomal karyotype AML also achieved marrow blast reductions of at least 50%.

### Everolimus trough level

**Figure 2:**
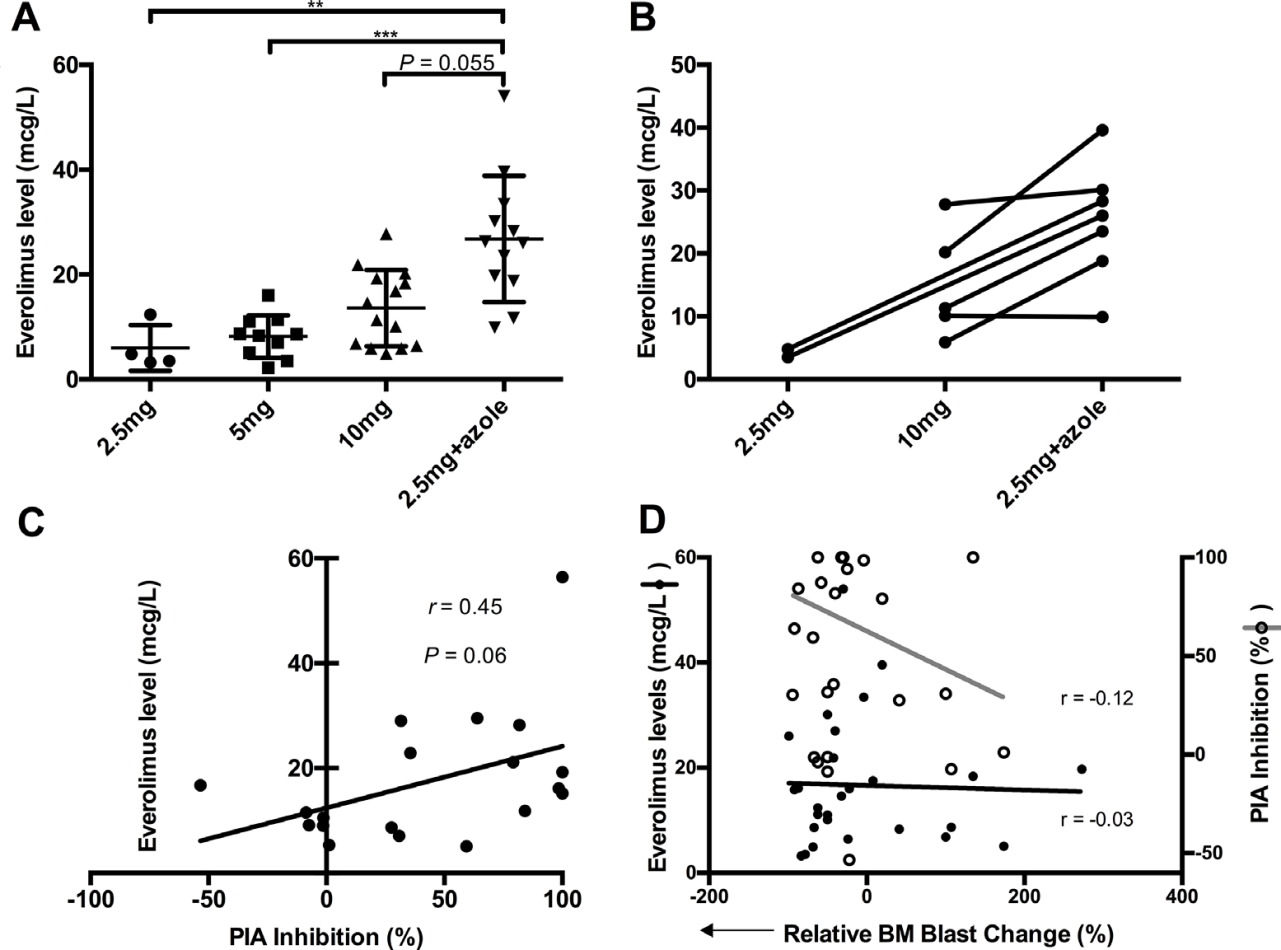
Everolimus pharmacokinetics and pharmacodynamics (**A**) Blood everolimus trough levels according to dose cohort. ** denotes *P* < 0.01 and *** denotes *P* < 0.001, adjusted for Dunn's multiple comparisons after a significant Kruskal-Wallis test. (**B**) Intra-patient changes in blood everolimus trough levels with the addition of azole and dose limited to 2.5 mg everolimus (data available for 7 patients). (**C**) Correlation of blood everolimus trough levels with Plasma Inhibitory Activity (PIA) for 19 patients. (**D**) Correlation of blood everolimus trough levels and PIA inhibition with the relative bone marrow blast reduction, respectively

Several patients switched from the allocated everolimus dose to the modified 2.5 mg dose when azoles were added. To gain insight into the unexpectedly superior outcomes observed in the everolimus/azole cohort, trough everolimus levels were examined. This revealed that everolimus levels were highest in the everolimus/azole cohort, even higher than patients administered everolimus 10 mg without azoles (Figure 2A). Substantial intra-patient increases in everolimus levels were demonstrated following the addition of antifungal azoles (Figure 2B). Furthermore, the cumulative everolimus dose was similar between the everolimus/azole cohort (median 394 mg) and those not receiving concurrent azoles (median 525 mg, *P* = 0.40).

**Figure 3:**
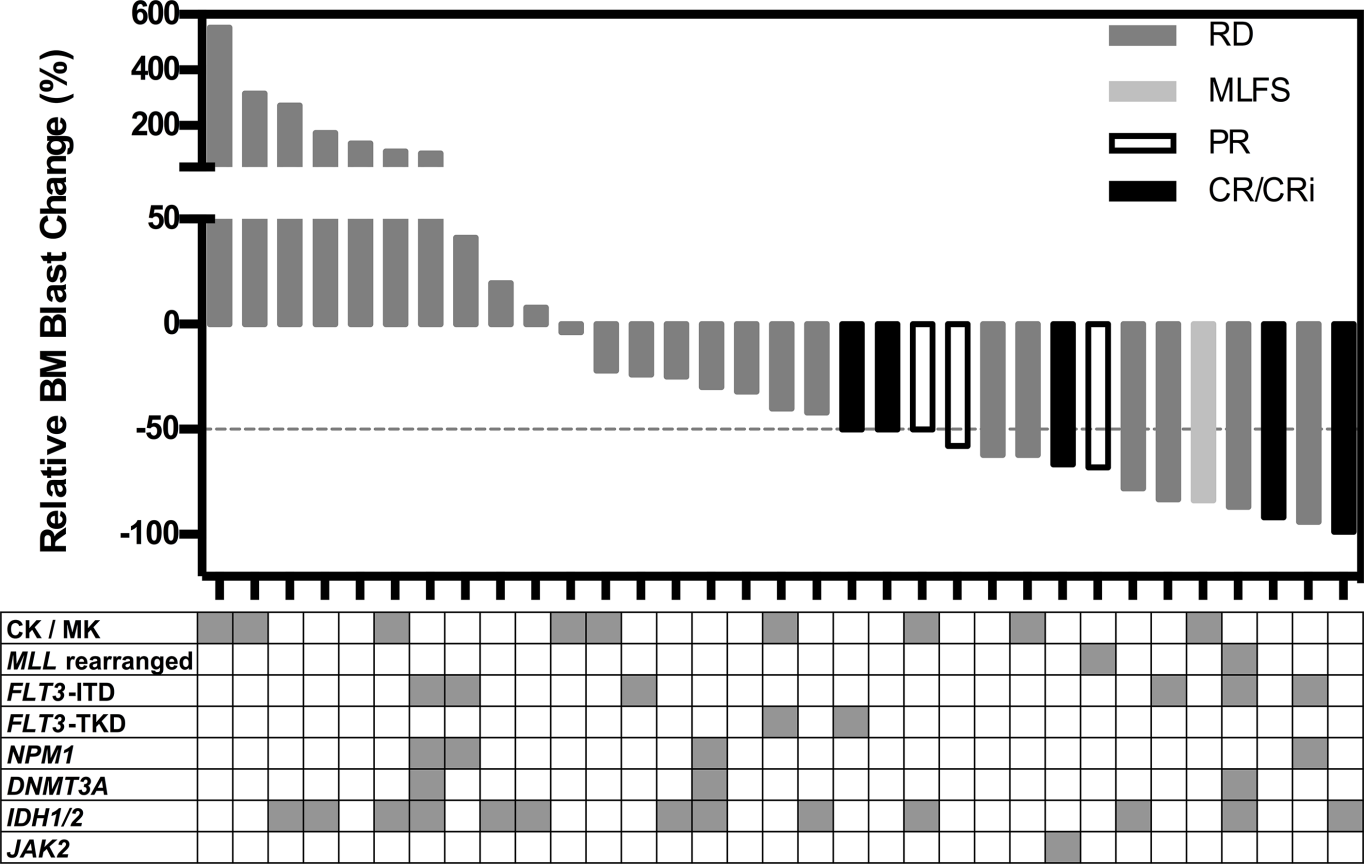
Waterfall plot of changes in relative bone marrow blast count in 33 patients Each vertical bar represents an individual patient, and the different greyscales represent the International Working Group response criteria. The table below shows the distribution of karyotypes and molecular genetics, with each column representing an individual patient, and the shaded area representing positive findings. *Abbreviations*: CK, complex karyotype; MK, monosomal karyotype.

### Plasma inhibitory activity (PIA) assays

To assess the pharmacodynamic activity of everolimus, a PIA assay was used to assess patient plasma collected on day 5 and 19 (prior to and after 2 weeks of everolimus therapy), to examine the potential for plasma to inhibit P-p70S6K in the OCI-AML3 reporter cell line (Supplementary Figure S1). PIA responses correlated with increasing everolimus levels recorded at the same time in a subset of patient samples that were available for analysis (Figure 2C). PIA responses also correlated with bone marrow blast reductions to a greater extent than everolimus drug levels (Figure 2D), though neither achieved statistical significance.

## DISCUSSION

The combination of everolimus with azacitidine was tolerable and deliverable to a group of predominantly older and heavily pre-treated patients with AML. Previous studies with hypomethylating agents have recorded response rates of 16–21% and OS outcomes of 6–9 months in older patients with relapsed/refractory AML [18–20]. In this study, the ORR was 22.5%, and median survival 8.5 months, consistent with previously reported outcomes for azacitidine in similar patient cohorts. The MTD was not identified for the combination of azacitidine with everolimus, despite escalating the everolimus dose to 10 mg daily from days 5–21.

In an attempt to facilitate the use of antifungal azoles in this patient population, everolimus was combined with voriconazole or posaconazole, with the concurrent everolimus dose reduced to 2.5 mg daily, due to the known pharmacokinetic interactions between these two drugs. Trough everolimus blood concentrations were noticeably higher among patients taking concomitant azoles, exemplified by the increased intra-patient concentrations (Figure 2), consistent with the known pharmacokinetic interaction with CYP3A4 [30–32]. Interestingly, landmark analysis beyond cycle 1 revealed that the everolimus/azole cohort had a higher ORR (50% *vs*. 16%, *P* = 0.056) and longer median OS (12.8 *vs*. 6.0 months, *P* = 0.049), when compared to patients not receiving concurrent azole, despite comparable everolimus doses delivered to each cohort. Fungal chest infection developed in 0/12 in the everolimus/azole cohort, and 2/19 in those without concurrent azole (*P* = 0.51). The correlation between superior response and drug exposure is consistent with another phase 1b study by Park *et al*. where everolimus was combined with conventional 7+3 chemotherapy in patients < 65 years of age at first relapse [15].

In our study, azacitidine and everolimus were given sequentially, similar to the sequential decitabine and rapamycin approach reported by Liesveld *et al*. [29], but in contrast to pulsed everolimus (up to 70 mg) on days 1 and 7 in combination with 7+3 chemotherapy [15]. Hypomethylating agents result in delayed demethylation, peaking 1–2 weeks after therapy. Sequential administration over 3 weeks was designed to maximise exposure of blasts to mTOR inhibition during the period of peak demethylation agent response. The schedule was also aimed at avoiding potential cell cycle inhibitory effects of mTOR inhibitors on azacitidine activity.

mTORC1 plays an important role in mRNA translation and autophagy regulation, whereas mTORC2 regulates the phosphorylation of AKT on Ser 473 (pAKT-S473) [33]. mTORC1 targeting by rapalogs could lead to unintended feedback amplification of the PI3K/AKT/mTOR pathway in AML, potentially subverting the clinical effect of targeting mTORC1 alone [4, 5]. Several studies have demonstrated improved anti-leukemic effects in AML by targeting both mTORC1 and mTORC2 simultaneously using active site or catalytic inhibitors [34]. Interestingly, mTORC2 may be inhibited by high doses of rapamycin, leading to inactivation of AKT and ERK [6]. Everolimus has also been reported to suppress mTORC2 and the AKT and ERK pathways in AML at doses approaching 20 nM [7], which is the concentration range achieved by the everolimus/azole combination in the current study (Figure 2). It is therefore possible that the superior outcome observed with the everolimus/azole combination is due to the inhibition of mTORC2, resulting from the higher blood concentrations of everolimus consequent to CYP3A4 inhibition by antifungal azoles. Although limitation of available patient samples prevented an analysis of pAKT-S473 in AML blasts among patients receiving everolimus/azole combination, this should be the objective of future validation studies.

Reliable molecular biomarkers of response to mTOR inhibitors remain to be determined. Ecotropic viral integration site 1 (*EVI1*) has been shown to directly repress *PTEN* transcription, resulting in increased sensitivity to rapamycin in *EVI1*-related mouse leukemia [35]. Poor prognosis karyotypes including monosomy 7 and t(11q23) are characterized by *EVI1* overexpression [36, 37]. Although 2/2 patients with *MLL-*rearranged AML in the current study had a > 50% reduction in bone marrow blasts, further studies will be required to determine the clinical significance of this association. Three patients with refractory *FLT3*-ITD AML had > 80% bone marrow blast reductions with everolimus/azacitidine therapy. Two of these patients went on to have allogeneic stem cell transplantation but subsequently relapsed and died. *FLT3*-ITD in AML is frequently found in combination with genomic lesions affecting epigenetic regulators [38], which may result in synergistic hypermethylation of genes, such as *GATA2* [39]. Future studies exploring azacitidine in combination with rapalogs should therefore include analysis of *FLT3*-ITD and mutations affecting epigenetic modifiers. Interestingly, *IDH1/2* mutation was considerably prevalent (15/35; 43%) in our predominantly relapsed and refractory study population, in contrast to the 16–21% observed in recently published large series of newly diagnosed AML [38, 40].

Although the MTD of everolimus (in combination with azacitidine) was not defined in this study, three patients developed delayed and unexpected cardiac dysfunction that only became apparent following allogeneic stem cell transplantation. All three patients received high-dose cyclophosphamide and high-dose total body irradiation (TBI) conditioning. Two other patients in the study allografted with either busulphan/cyclophosphamide or low-dose TBI conditioning regimen did not develop subsequent cardiac compromise. The pre-transplant cardiac exposure to mTOR inhibitors followed by TBI is uncommon, so these three cardiac events should promote caution for future studies among patients likely to receive potent mTOR inhibitors in combination with azacitidine prior to irradiation therapy involving the heart. Disruption of cardiac mTORC1 is associated with a significant reduction in cardiomyocytes and cardiac dilation in mice, suggesting an important role of mTOR in cardiomyocyte survival during development [41, 42]. mTOR inhibition may decrease the apoptotic threshold and enhance autophagy, leading to hypersensitivity of tissues to radiotherapy in patients treated with a mTOR inhibitor [43]. In our study, this scenario may have been further exacerbated by the frequent exposure of our patient population to prior anthracyclines.

In summary, the combination of everolimus and azacitidine is a safe and well-tolerated treatment for relapsed and refractory AML. Future studies should explore the potentially beneficial pharmacokinetic interaction between everolimus and antifungal azoles in delivering higher everolimus concentrations in combination with azacitidine *in vivo*, and to examine the impact this might have on the regulation of mTORC1 and mTORC2-mediated pathways in AML blasts. Further studies are also warranted to define the potential for *FLT3*-ITD, *MLL* rearrangement and other genomic markers to predict response to this therapy. In addition, our results also support the clinical investigation of hypomethylating agents in combination with newer generation dual mTORC1/mTORC2 inhibitors [44, 45] or compound PI3K/mTOR inhibitors [46, 47] in patients with AML.

## MATERIALS AND METHODS

### Patients

Eligible patients were relapsed or refractory AML who had received up to two prior lines of intensive therapy, and untreated AML in patients aged ≥ 60 years unfit for intensive chemotherapy. A full list of eligibility criteria is detailed in Supplementary Table S1.

### Study design and treatment

This open-label, phase Ib/II study was conducted at the Alfred Hospital, approved by an independent Human Research and Ethics Committee and registered with the Australian and New Zealand Clinical Trials Registry (ACTRN12610001031055). Azacitidine 75 mg/m^2^ was injected subcutaneously on days 1–5 and 8–9 [48]. The everolimus dose was escalated in a standard 3 × 3 study design to determine the MTD. The cohort doses of everolimus examined were 2.5, 5 and 10 mg with no intra-patient dose escalation permitted; the dosage was capped at 10 mg as this was the established dose used in the treatment of solid cancers [49]. After establishment of the MTD, a dose expansion group was included to better estimate the tolerability of this dose and to provide preliminary assessment of efficacy. Due to the CYP3A4 interaction between everolimus and antifungal azoles, azole administration was excluded from the first cycle of therapy, but permitted (voriconazole or posaconazole) in subsequent cycles with the dose of everolimus limited to 2.5 mg. Everolimus was administered orally daily on days 5–21 of each 28–day cycle. Hydroxyurea was permitted up to 48 hours preceding commencement of study medication, but no other anti-leukemic therapies were permitted. Patients were permitted to receive transfusion support in accordance with established guidelines along with granulocyte colony stimulating factor (G-CSF) and prophylactic antimicrobials at physician discretion. Patients were continued on therapy until AML relapse or progression, unacceptable toxicity or completion of study related procedures.

### Objectives and endpoints

The main objective was to examine the safety and tolerability of everolimus in combination with azacitidine in relapsed/refractory AML. Secondary objectives were to provide preliminary data regarding clinical outcomes and the correlation between clinical responses and exploratory biomarkers of outcome.

### Assessments

#### Toxicity

Adverse events were graded according to the National Cancer Institute CTCAE version 4. DLT was defined as treatment-related Grade 3–4 non-hematologic toxicity attributable to either study drug, or persistent grade 4 neutropenia or thrombocytopenia by day 42 of first cycle with bone marrow cellularity < 10% not related to persistent leukemia (bone marrow blasts < 5%). Patients were evaluable for toxicity if ≥ 85% of cycle 1 therapy was completed, or if the early withdrawal was toxicity-related. Patients with early withdrawal due to disease progression were excluded from the safety analysis, but included for the efficacy analysis.

### Efficiancy

Response criteria were graded as per the IWG criteria [50]. OS was calculated from first day of study therapy to date of death. RFS was calculated from date of confirmed response, inclusive of CR, CRi, MLFS and PR, to the date of confirmed relapse or loss of PR. Time-to-event analyses were censored for patients who underwent transplant or lost to follow-up.

### Correlative assessments

Everolimus trough blood levels were measured by liquid chromatography mass spectrometry ∼day 19, two weeks after the commencement of the mTOR inhibitor. Pharmacodynamic assessment of everolimus activity was by PIA of P-p70S6K levels by Western blot using the OCI-AML3 cell line, with plasma taken on days 5 and 19. Briefly, frozen plasma samples were thawed, clarified by centrifugation, and washed twice with ice-cold phosphate-buffered saline prior to cell lysis. After immunoblotting for phosphorylated p70S6K, densitometric analysis was performed on the bands, and the PIA for each plasma sample was calculated by expressing the density of its corresponding band as a percentage of the density of the baseline band (which was arbitrarily set at 100%).

### Molecular genetic testing

*FLT3-*ITD detection was by PCR followed by fragment analysis using capillary electrophoresis [51]. Bone marrow samples at baseline were also assessed for the presence of mutations in *c-KIT*, *DNMT3A*, *FLT3-*TKD, *IDH1*, *IDH2*, *JAK1*, *JAK2*, *MPL*, *NPM1*, *KRAS* and *NRAS* by multiplexed mass spectrometry (MassARRAY System, Sequenom, San Diego, CA, USA), as previously described [52].

### Statistical analysis

Statistical analysis was performed using Graphpad Prism version 6 (GraphPad Software, La Jolla, California, USA) and the R statistical software version 3.2.3 (R foundation for statistical computing, Vienna, Austria). Survival curves were calculated by the Kaplan-Meier method and the log-rank test was used to compare patient groups. A *χ*^2^ test and Fisher's exact test was used to compare correlative response outcomes.

## SUPPLEMENTARY TABLES AND FIGURE



## Abbreviations

4EBP1: Eukaryotic translation initiation factor 4E-binding protein 1
AKT: Protein kinase B
AML: Acute myeloid leukemia
Cmax: Maximum concentration
CR: Complete remission
CRi: Complete remission with incomplete blood count recovery
CTCAE: Common terminology criteria for adverse events
CYP3A4: Cytochrome P450 3A4
DLT: Dose limiting toxicity
ERK: Extracellular signal-regulated kinases
*EVI1*: Ecotropic viral integration site 1
*FLT3*-ITD: Fms like tyrosine kinase 3 internal tandem duplications
*GATA2*: GATA binding protein 2
IWG: International working group
MLFS: Morphologic leukemia-free state
*MLL*: Mixed lineage leukemia
mRNA: Messenger ribonucleic acid
MTD: Maximum tolerated dose
mTOR: Mammalian target of rapamycin
mTORC1: Mammalian target of rapamycin complex 1
mTORC2: Mammalian target of rapamycin complex 2
ORR: Overall response rate
OS: Overall survival
p70S6K: 70 kDa ribosomal S6 kinase
pAKT-S473: Phosphorylation of AKT on Ser 473
P-p70S6K: Phosphorylation of 70 kDa ribosomal S6 kinase
PI3K: Phosphoinositide 3-kinase
PIA: Plasma inhibitory activity
*PPP2R2B*: Protein phosphatase 2 regulatory subunit B, beta isoform
PR: Partial remission
*PTEN*: Phosphatase and tensin homolog
RFS: Relapse-free survival
TBI: Total body irradiation
*TSC2*: Tuberous sclerosis complex 2

## References

[1] Xu Q, Simpson SE, Scialla TJ, Bagg A, Carroll M (2003). Survival of acute myeloid leukemia cells requires PI3 kinase activation. Blood.

[2] Park S, Chapuis N, Tamburini J, Bardet V, Cornillet-Lefebvre P, Willems L, Green A, Mayeux P, Lacombe C, Bouscary D (2010). Role of the PI3K/AKT and mTOR signaling pathways in acute myeloid leukemia. Haematologica.

[3] Min YH, Eom JI, Cheong JW, Maeng HO, Kim JY, Jeung HK, Lee ST, Lee MH, Hahn JS, Ko YW (2003). Constitutive phosphorylation of Akt/PKB protein in acute myeloid leukemia: its significance as a prognostic variable. Leukemia.

[4] Wan X, Harkavy B, Shen N, Grohar P, Helman LJ (2007). Rapamycin induces feedback activation of Akt signaling through an IGF-1R-dependent mechanism. Oncogene.

[5] Tamburini J, Chapuis N, Bardet V, Park S, Sujobert P, Willems L, Ifrah N, Dreyfus F, Mayeux P, Lacombe C, Bouscary D (2008). Mammalian target of rapamycin (mTOR) inhibition activates phosphatidylinositol 3-kinase/Akt by up-regulating insulin-like growth factor-1 receptor signaling in acute myeloid leukemia: rationale for therapeutic inhibition of both pathways. Blood.

[6] Chen XG, Liu F, Song XF, Wang ZH, Dong ZQ, Hu ZQ, Lan RZ, Guan W, Zhou TG, Xu XM, Lei H, Ye ZQ, Peng EJ (2010). Rapamycin regulates Akt and ERK phosphorylation through mTORC1 and mTORC2 signaling pathways. Mol Carcinog.

[7] Zeng Z, Sarbassov dos D, Samudio IJ, Yee KW, Munsell MF, Ellen Jackson C, Giles FJ, Sabatini DM, Andreeff M, Konopleva M (2007). Rapamycin derivatives reduce mTORC2 signaling and inhibit AKT activation in AML. Blood.

[8] Recher C, Beyne-Rauzy O, Demur C, Chicanne G, Dos Santos C, Mas VM, Benzaquen D, Laurent G, Huguet F, Payrastre B (2005). Antileukemic activity of rapamycin in acute myeloid leukemia. Blood.

[9] Yilmaz OH, Valdez R, Theisen BK, Guo W, Ferguson DO, Wu H, Morrison SJ (2006). Pten dependence distinguishes haematopoietic stem cells from leukaemia-initiating cells. Nature.

[10] Hoshii T, Tadokoro Y, Naka K, Ooshio T, Muraguchi T, Sugiyama N, Soga T, Araki K, Yamamura K, Hirao A (2012). mTORC1 is essential for leukemia propagation but not stem cell self-renewal. J Clin Invest.

[11] Yee KW, Zeng Z, Konopleva M, Verstovsek S, Ravandi F, Ferrajoli A, Thomas D, Wierda W, Apostolidou E, Albitar M, O’Brien S, Andreeff M, Giles FJ (2006). Phase I/II study of the mammalian target of rapamycin inhibitor everolimus (RAD001) in patients with relapsed or refractory hematologic malignancies. Clin Cancer Res.

[12] Rizzieri DA, Feldman E, Dipersio JF, Gabrail N, Stock W, Strair R, Rivera VM, Albitar M, Bedrosian CL, Giles FJ (2008). A phase 2 clinical trial of deforolimus (AP23573, MK-8669), a novel mammalian target of rapamycin inhibitor, in patients with relapsed or refractory hematologic malignancies. Clin Cancer Res.

[13] Boehm A, Mayerhofer M, Herndlhofer S, Knoebl P, Sillaber C, Sperr WR, Jaeger U, Valent P (2009). Evaluation of in vivo antineoplastic effects of rapamycin in patients with chemotherapy-refractory AML. Eur J Intern Med.

[14] Callera F, Lopes CO, Rosa ES, Mulin CC (2008). Lack of antileukemic activity of rapamycin in elderly patients with acute myeloid leukemia evolving from a myelodysplastic syndrome. Leuk Res.

[15] Park S, Chapuis N, Saint Marcoux F, Recher C, Prebet T, Chevallier P, Cahn JY, Leguay T, Bories P, Witz F, Lamy T, Mayeux P, Lacombe C (2013). A phase Ib GOELAMS study of the mTOR inhibitor RAD001 in association with chemotherapy for AML patients in first relapse. Leukemia.

[16] Perl AE, Kasner MT, Tsai DE, Vogl DT, Loren AW, Schuster SJ, Porter DL, Stadtmauer EA, Goldstein SC, Frey NV, Nasta SD, Hexner EO, Dierov JK (2009). A phase I study of the mammalian target of rapamycin inhibitor sirolimus and MEC chemotherapy in relapsed and refractory acute myelogenous leukemia. Clin Cancer Res.

[17] Amadori S, Stasi R, Martelli AM, Venditti A, Meloni G, Pane F, Martinelli G, Lunghi M, Pagano L, Cilloni D, Rossetti E, Di Raimondo F, Fozza C (2012). Temsirolimus, an mTOR inhibitor, in combination with lower-dose clofarabine as salvage therapy for older patients with acute myeloid leukaemia: results of a phase II GIMEMA study (AML-1107). Br J Haematol.

[18] Itzykson R, Thepot S, Berthon C, Delaunay J, Bouscary D, Cluzeau T, Turlure P, Prebet T, Dartigeas C, Marolleau JP, Recher C, Plantier I, Stamatoullas A (2015). Azacitidine for the treatment of relapsed and refractory AML in older patients. Leuk Res.

[19] Ivanoff S, Gruson B, Chantepie SP, Lemasle E, Merlusca L, Harrivel V, Charbonnier A, Votte P, Royer B, Marolleau JP (2013). 5-Azacytidine treatment for relapsed or refractory acute myeloid leukemia after intensive chemotherapy. Am J Hematol.

[20] Ritchie EK, Feldman EJ, Christos PJ, Rohan SD, Lagassa CB, Ippoliti C, Scandura JM, Carlson K, Roboz GJ (2013). Decitabine in patients with newly diagnosed and relapsed acute myeloid leukemia. Leuk Lymphoma.

[21] Sun D, Toan X, Zhang Y, Chen Y, Lu R, Wang X, Fang J (2008). Mammalian target of rapamycin pathway inhibition enhances the effects of 5-aza-dC on suppressing cell proliferation in human gastric cancer cell lines. Sci China C Life Sci.

[22] Zhang YJ, Zhao SL, Tian XQ, Sun DF, Xiong H, Dai Q, Li XQ, Fang JY (2009). Combined inhibition of Dnmt and mTOR signaling inhibits formation and growth of colorectal cancer. Int J Colorectal Dis.

[23] Qian XJ, Li YT, Yu Y, Yang F, Deng R, Ji J, Jiao L, Li X, Wu RY, Chen WD, Feng GK, Zhu XF (2015). Inhibition of DNA methyltransferase as a novel therapeutic strategy to overcome acquired resistance to dual PI3K/mTOR inhibitors. Oncotarget.

[24] Chakraborty S, Mohiyuddin SM, Gopinath KS, Kumar A (2008). Involvement of TSC genes and differential expression of other members of the mTOR signaling pathway in oral squamous cell carcinoma. BMC Cancer.

[25] Liao H, Xiao Y, Hu Y, Xiao Y, Yin Z, Liu L, Kang X, Chen Y (2016). Methylation-induced silencing of miR-34a enhances chemoresistance by directly upregulating ATG4B-induced autophagy through AMPK/mTOR pathway in prostate cancer. Oncol Rep.

[26] Liesveld JL, Rosell K, Lu C, Mulford D, Walker A (2007). The mTOR Inhibitor Rapamycin Demonstrates Activity Against AML in Combination with Imatinib Mesylate and with 5-Azacytidine. Blood.

[27] Xu Z, Wang M, Wang L, Wang Y, Zhao X, Rao Q, Wang J (2009). Aberrant expression of TSC2 gene in the newly diagnosed acute leukemia. Leuk Res.

[28] Nishioka C, Ikezoe T, Yang J, Koeffler HP, Yokoyama A (2008). Blockade of mTOR signaling potentiates the ability of histone deacetylase inhibitor to induce growth arrest and differentiation of acute myelogenous leukemia cells. Leukemia.

[29] Liesveld JL, O’Dwyer K, Walker A, Becker MW, Ifthikharuddin JJ, Mulford D, Chen R, Bechelli J, Rosel K, Minhajuddin M, Jordan CT, Phillips GL (2013). A phase I study of decitabine and rapamycin in relapsed/refractory AML. Leuk Res.

[30] Kovarik JM, Beyer D, Bizot MN, Jiang Q, Shenouda M, Schmouder RL (2005). Blood concentrations of everolimus are markedly increased by ketoconazole. J Clin Pharmacol.

[31] Billaud EM, Antoine C, Berge M, Abboud I, Lefeuvre S, Benammar M, Glotz D (2009). Management of metabolic cytochrome P450 3A4 drug-drug interaction between everolimus and azole antifungals in a renal transplant patient. Clin Drug Investig.

[32] Outeda Macias M, Salvador Garrido P, Elberdin Pazos L, Martin Herranz MI (2016). Management of Everolimus and Voriconazole Interaction in Lung Transplant Patients. Ther Drug Monit.

[33] Bhaskar PT, Hay N (2007). The two TORCs and Akt. Dev Cell.

[34] Carneiro BA, Kaplan JB, Altman JK, Giles FJ, Platanias LC (2015). Targeting mTOR signaling pathways and related negative feedback loops for the treatment of acute myeloid leukemia. Cancer Biol Ther.

[35] Yoshimi A, Goyama S, Watanabe-Okochi N, Yoshiki Y, Nannya Y, Nitta E, Arai S, Sato T, Shimabe M, Nakagawa M, Imai Y, Kitamura T, Kurokawa M (2011). Evi1 represses PTEN expression and activates PI3K/AKT/mTOR via interactions with polycomb proteins. Blood.

[36] Ho PA, Alonzo TA, Gerbing RB, Pollard JA, Hirsch B, Raimondi SC, Cooper T, Gamis AS, Meshinchi S (2013). High EVI1 expression is associated with MLL rearrangements and predicts decreased survival in paediatric acute myeloid leukaemia: a report from the children’s oncology group. Br J Haematol.

[37] Lugthart S, van Drunen E, van Norden Y, van Hoven A, Erpelinck CA, Valk PJ, Beverloo HB, Lowenberg B, Delwel R (2008). High EVI1 levels predict adverse outcome in acute myeloid leukemia: prevalence of EVI1 overexpression and chromosome 3q26 abnormalities underestimated. Blood.

[38] Papaemmanuil E, Gerstung M, Bullinger L, Gaidzik VI, Paschka P, Roberts ND, Potter NE, Heuser M, Thol F, Bolli N, Gundem G, Van Loo P, Martincorena I (2016). Genomic Classification and Prognosis in Acute Myeloid Leukemia. N Engl J Med.

[39] Shih AH, Jiang Y, Meydan C, Shank K, Pandey S, Barreyro L, Antony-Debre I, Viale A, Socci N, Sun Y, Robertson A, Cavatore M, de Stanchina E (2015). Mutational cooperativity linked to combinatorial epigenetic gain of function in acute myeloid leukemia. Cancer Cell.

[40] Metzeler KH, Herold T, Rothenberg-Thurley M, Amler S, Sauerland MC, Görlich D, Schneider S, Konstandin NP, Dufour A, Bräundl K, Ksienzyk B, Zellmeier E, Hartmann L (2016). Spectrum and prognostic relevance of driver gene mutations in acute myeloid leukemia. Blood.

[41] Zhang D, Contu R, Latronico MV, Zhang J, Rizzi R, Catalucci D, Miyamoto S, Huang K, Ceci M, Gu Y, Dalton ND, Peterson KL, Guan KL (2010). MTORC1 regulates cardiac function and myocyte survival through 4E-BP1 inhibition in mice. J Clin Invest.

[42] Zhang P, Shan T, Liang X, Deng C, Kuang S (2014). Mammalian target of rapamycin is essential for cardiomyocyte survival and heart development in mice. Biochem Biophys Res Commun.

[43] Kim KW, Hwang M, Moretti L, Jaboin JJ, Cha YI, Lu B (2008). Autophagy upregulation by inhibitors of caspase-3 and mTOR enhances radiotherapy in a mouse model of lung cancer. Autophagy.

[44] Zeng Z, Shi YX, Tsao T, Qiu Y, Kornblau SM, Baggerly KA, Liu W, Jessen K, Liu Y, Kantarjian H, Rommel C, Fruman DA, Andreeff M (2012). Targeting of mTORC1/2 by the mTOR kinase inhibitor PP242 induces apoptosis in AML cells under conditions mimicking the bone marrow microenvironment. Blood.

[45] Altman JK, Sassano A, Kaur S, Glaser H, Kroczynska B, Redig AJ, Russo S, Barr S, Platanias LC (2011). Dual mTORC2/mTORC1 targeting results in potent suppressive effects on acute myeloid leukemia (AML) progenitors. Clin Cancer Res.

[46] Chapuis N, Tamburini J, Green AS, Vignon C, Bardet V, Neyret A, Pannetier M, Willems L, Park S, Macone A, Maira SM, Ifrah N, Dreyfus F (2010). Dual inhibition of PI3K and mTORC1/2 signaling by NVP-BEZ235 as a new therapeutic strategy for acute myeloid leukemia. Clin Cancer Res.

[47] Colamonici M, Blyth G, Saleiro D, Szilard A, Bliss-Moreau M, Giles FJ, Altman JK, Beauchamp EM, Platanias LC (2015). Dual targeting of acute myeloid leukemia progenitors by catalytic mTOR inhibition and blockade of the p110alpha subunit of PI3 kinase. Oncotarget.

[48] Lyons RM, Cosgriff TM, Modi SS, Gersh RH, Hainsworth JD, Cohn AL, McIntyre HJ, Fernando IJ, Backstrom JT, Beach CL (2009). Hematologic response to three alternative dosing schedules of azacitidine in patients with myelodysplastic syndromes. J Clin Oncol.

[49] Everolimus. Lexicomp Online®.

[50] Cheson BD, Bennett JM, Kopecky KJ, Buchner T, Willman CL, Estey EH, Schiffer CA, Doehner H, Tallman MS, Lister TA, Lo-Coco F, Willemze R, Biondi A (2003). Revised recommendations of the International Working Group for Diagnosis, Standardization of Response Criteria, Treatment Outcomes, and Reporting Standards for Therapeutic Trials in Acute Myeloid Leukemia. J Clin Oncol.

[51] Murphy KM, Levis M, Hafez MJ, Geiger T, Cooper LC, Smith BD, Small D, Berg KD (2003). Detection of FLT3 internal tandem duplication and D835 mutations by a multiplex polymerase chain reaction and capillary electrophoresis assay. J Mol Diagn.

[52] Tan P, Wei A, Mithraprabhu S, Cummings N, Liu HB, Perugini M, Reed K, Avery S, Patil S, Walker P, Mollee P, Grigg A, D’Andrea R (2014). Dual epigenetic targeting with panobinostat and azacitidine in acute myeloid leukemia and high-risk myelodysplastic syndrome. Blood Cancer J.

