# Supplementary material for: The mTOR inhibitor everolimus in combination with azacitidine in patients with relapsed/refractory acute myeloid leukemia: a phase Ib/II study
Source: Oncotarget. 2016 Nov 29;8(32):52269–80. doi: 10.18632/oncotarget.13699 (PMC5581027; doi:10.18632/oncotarget.13699)
Supplement: Supplementary file 1 [file oncotarget-08-52269-s001.pdf]

The mTOR inhibitor everolimus in combination with azacitidine in patients with relapsed/refractory acute myeloid leukemia: a phase Ib/II study

Supplementary Materials

Supplementary Table S1: Inclusion & exclusion criteria

Inclusion criteria

- Over the age of 18 who have received up to 2 previous lines of intensive chemotherapy
- No prior failure to achieve at least a PR with Azacitidine or Everolimus
- Provision of written informed consent
- Secondary AML (including therapy-related) are included
- Life expectancy of greater than 3 months in relation to diseases other than AML/MDS
- ECOG performance status 0 – 3
- Electrolyte levels (potassium, calcium (albumin-adjusted), magnesium, phosphorous) within normal limits (WNL) or easily correctable with supplements
- Adequate hepatic function as defined by bilirubin ≤ 1.5 × the upper limit of normal (ULN) and aspartate aminotransferase (AST) and alanine aminotransferase (ALT) ≤ 2.5 × ULN
- Adequate renal function, with serum creatinine ≤ 1.5 × ULN or GFR > 30 ml/minute
- Patients with no uncontrolled active infection
- Hydroxyurea ceased 48 hours prior to study therapy

Exclusion criteria

- Any serious medical or psychiatric conditions which the investigator feels may interfere with the patient's ability to give informed consent or participate in the procedures or evaluations of the study
- History of major non-compliance to medication
- Evidence of CNS leukaemia
- Uncontrolled viral infection with known HIV or Hepatitis type B or C
- Currently active gastrointestinal disease (e.g., ulcerative diseases, uncontrolled nausea, vomiting, diarrhoea, malabsorption syndrome, or small bowel resection), or other disease, that prevents the patient from absorbing or taking oral medication
- Any other concurrent severe and/or uncontrolled medical conditions (e.g. acute or chronic liver disease, infection, pulmonary disease) that in the opinion of the investigator could potentiate unacceptable safety risks or jeopardize compliance with the protocol
- Males with a female partner of childbearing potential do not agree to use at least 2 effective contraceptive methods throughout the study and for 6 months following the date of last dose

Supplementary Table S2: Haematological toxicities (all cycles) unrelated to pre-existing cytopenias

Haematological toxiciwwties (evaluable *n*)		Grade 3	Grade 4	Total	%
**Anaemia**	(31)	19	2	21	67.7
**Neutropenia**	(15)	1	10	11	73.3
**Thrombocytopenia**	(21)	3	11	14	66.6

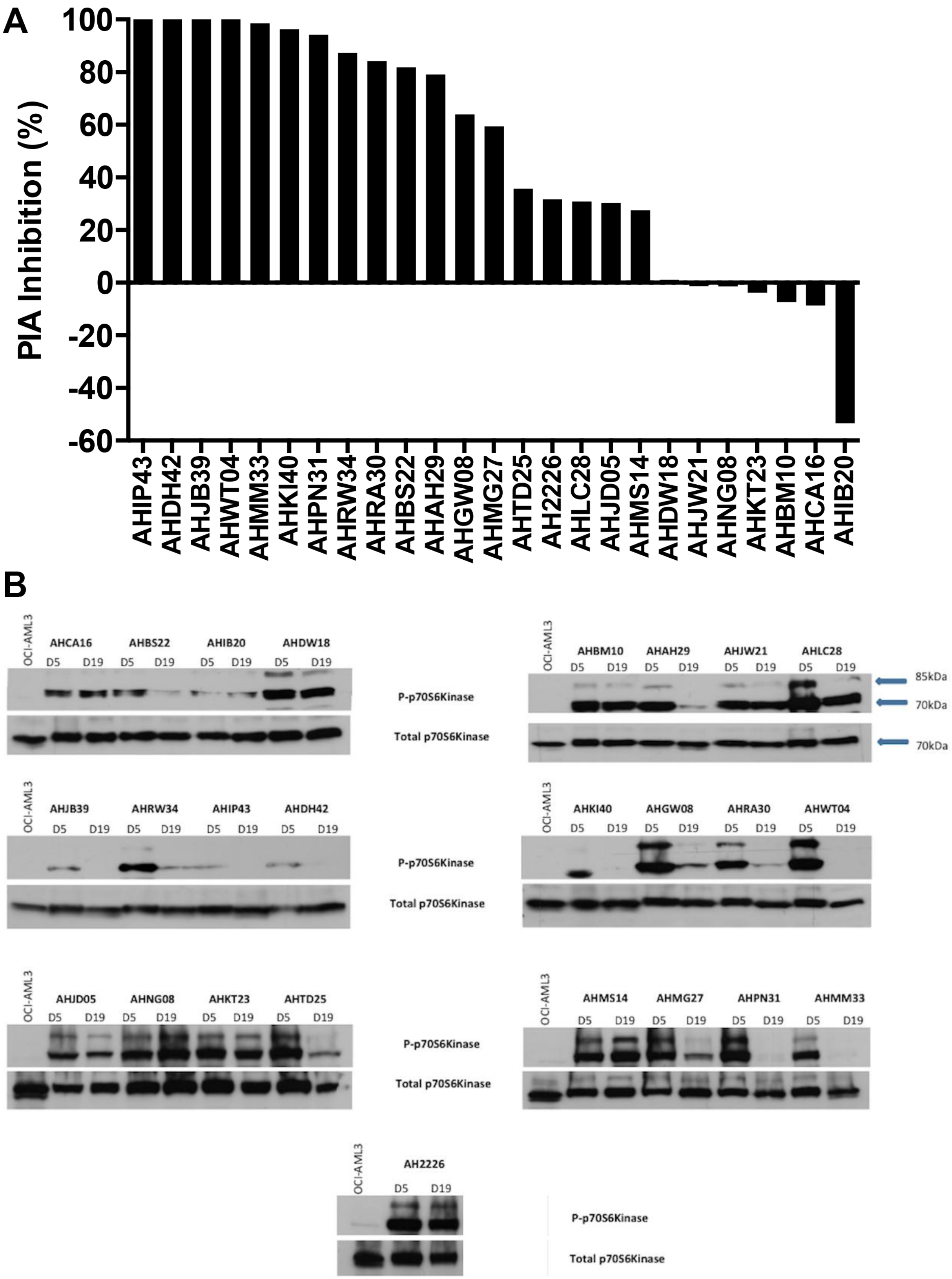

Supplementary Figure S1: Pharmacodynamic response as measured by plasma inhibitory activity (PIA) assay in 25 patients. OCI-AML3 cell lines were exposed to patient serum. P-p70S6 kinase levels at day 19 were expressed as the percentage (%) of the levels at day 5, as quantitated by densitometry (**A**) based on primary blots shown in (**B**). A duplicated paired immunoblot experiment has been deleted for subject AHAH29, in whom an averaged value is used for plotting.
